# Occlusion Effect in Response to Stimulation by Soft Tissue Conduction-Implications ^†^

**DOI:** 10.3390/audiolres10020012

**Published:** 2020-12-04

**Authors:** Miriam Geal-Dor, Cahtia Adelman, Shai Chordekar, Haim Sohmer

**Affiliations:** 1 Speech & Hearing Center, Hebrew University-Hadassah Medical Center, Jerusalem 91200, Israel; gmiriam@hadassah.org.il (M.G.-D.); cahtiaa@hadassah.org.il (C.A.); 2 Department of Communication Disorders, Hadassah Academic College, Jerusalem 91200, Israel; shaychor@gmail.com; 3 Department of Communication Disorders, Sackler Faculty of Medicine, Tel Aviv University, Tel Aviv-Yafo 60198, Israel; 4 Department of Medical Neurobiology, Hebrew University-Hadassah Medical School, P.O. Box 12272, Jerusalem 91120, Israel

**Keywords:** bone conduction, soft tissue conduction, Bekesy, stethoscope, occlusion effect

## Abstract

To gain insight into the broader implications of the occlusion effect (OE—difference between unoccluded and occluded external canal thresholds), the OE in response to pure tones at 0.5, 1.0, 2.0 and 4.0 kHz to two bone conduction sites (mastoid and forehead) and two soft tissue conduction (STC) sites (under the chin and at the neck) were assessed. The OE was present at the soft tissue sites and at the bone conduction sites, with no statistical difference between them. The OE was significantly greater at lower frequencies, and negligible at higher frequencies. It seems that the vibrations induced in the soft tissues (STC) during stimulation at the soft tissue sites are conducted not only to the inner ear and elicit hearing, but also reach the walls of the external canal and initiate air pressures in the occluded canal which drive the tympanic membrane and excite the inner ear, leading to hearing. Use of a stethoscope by the internist to hear intrinsic body sounds (heartbeat, blood flow) serves as a clear demonstration of STC and its relation to hearing.

## 1. Introduction

The most common mode of hearing is by air conduction (AC)—i.e., by alternating condensation and rarefaction air pressures arising from the sound producing structure (e.g., natural voice, loud speaker, musical instrument, etc.), which are conducted to the ear, reaching the tympanic membrane, causing its vibration, and that of the middle ear ossicles, inner ear fluid pressures, etc. For clinical diagnostic purposes, bone conduction (BC) stimulation is elicited by a vibrator applied to skull bone, usually the skin over the mastoid or forehead, and it is used to differentiate between a conductive hearing loss (in which AC thresholds are elevated and BC thresholds are normal) and a sensori-neural hearing loss (in which both AC and BC thresholds are elevated). This differentiation was based on the assumption that BC stimulation excites the inner ear directly. It is now apparent that hearing can also be initiated by the vibrations induced in the soft tissues of the body by applying the clinical bone vibrator to sites on the body not over skull bone, such as the neck or under the chin, or are induced naturally, intrinsically in the body by self-vocalizations or by the heartbeat and blood flow. The vibrations induced are conducted by the soft tissues (therefore referred to as soft tissue conduction—STC) of the body to the body surface, and at the same time, to the ear, eliciting hearing [1]. Following the description of STC, the term “osseous BC” is applied to those forms of BC which are derived from the induction of actual vibrations of skull bone, leading to the vibration of the bone of the outer ear (occlusion effect), of the middle ear (ossicle inertia) and of the inner ear (fluid inertia and distortion) [2]. A special bone vibrator eliciting osseous BC is also used as a hearing aid in some cases of hearing loss (bone anchored hearing aid).

Following the elucidation of STC, the present study was designed to assess and compare the thresholds of normal hearing participants in response to several frequencies of vibratory stimuli delivered by a clinical bone vibrator to two soft tissue sites (submental region under the chin, and to the neck—over the sterno-cleido-mastoid muscle) and also to two osseous BC sites (mastoid and forehead). The thresholds were determined when their auditory canal was open (unoccluded) and when occluded with a foam earplug that provides 30 dB attenuation. The difference between unoccluded and occluded thresholds could then be calculated. Such a value would represent the magnitude of the occlusion effect (OE), defined as the enhancement of the threshold to BC resulting from occlusion of the external auditory canal [3]. The OE is thought to derive from vibrations of the wall of the external canal induced by the BC stimulation, producing air pressures in the canal. In the open canal condition, the air pressures for the most part are dispersed (i.e., dissipated) into the surroundings. However, when the canal is occluded, the entrapped air pressures in the canal sum with the vibrations of the tympanic membrane, ossicular chain, etc., as in AC, enhance the response of the inner ear.

The purpose of the present study was to assess the magnitude of the OE to STC stimulation, and to compare it to that to more conventional BC stimulation in order to gain further insight into the broader implications of STC stimulation.

## 2. Methods

Ten normal hearing female subjects (mean ± SD age 34.6 ± 11.7; range 25 to 55 years) (defined as AC thresholds at or better than 15 dB HL at the frequencies 0.5, 1.0, 2.0 and 4.0 kHz) participated in the study and comprised the students and staff of a college for women. All auditory testing was conducted using the same clinical audiometer (Interacoustics model AC40, Assens, Denmark), in the same audiometric booth, by the same audiologist. The participants were not aware of the intention of the study. They gave their written informed consent.

The participants underwent a conventional audiometric evaluation (AC thresholds conducted with warble tones, as is standard in our facility) by an audiologist in the conventional manner (ANSI S3.31 1978, 1986), using the modified Hughson-Westlake technique. AC threshold was determined using TDH 39 earphones (Telephonics Corp., Farmingdale, NY, USA). BC threshold was determined with the clinical bone vibrator (Radioear B71, New Eagle, PA, USA) applied to the classical BC sites on the right mastoid and forehead. In addition, the threshold was determined by applying the same bone vibrator to STC sites: the submental area (under the chin), as well as the skin over the right sterno-cleido-mastoid (SCM) muscle (neck site). With respect to the submental site, the subjects were instructed to avoid contact between the teeth of the upper jaw with those of the lower jaw, and this was verified by the experimenter. These sites were chosen due to their convenience and previous experience with these sites [4]. Originally, we had intended to apply the bone vibrator to each site with a spring calibrated to deliver an application force of 500 g (5 Newton), as in previous studies. However, it was inconvenient to use the spring at several of the sites, so instead, the bone vibrator was pressed against each of the four stimulation sites with a RadioEar Amband elastic headband (Denmark), usually used to deliver stimulation by a bone conductor hearing aid. The length of elastic headband could be adjusted with “Velcro” hook and loop fasteners, so that the stretching of the band could fit the different stimulation sites and head sizes. In this way, the application force was approximately the same for all participants, sites and conditions (occluded and unoccluded). In one subject, we succeeded in delivering the stimuli both with the 5 Newton spring and with the elastic band, and similar thresholds were obtained.

The thresholds to stimulation at the four sites were measured twice—once with the external ear canal open, and once with the canal occluded by a foam ear plug (Classic SuperFit 30 AeroCo, E-A-R Indianapolis, IN, USA) which was first rolled to make it narrower, and then inserted into the ear canal to provide 30 dB attenuation. The contralateral left ear was masked by presenting appropriate 40 dB SL narrow band masking noise in all conditions by a TDH 39 earphone. Using this experimental procedure, eight threshold audiograms were conducted in response to stimulation at 0.5, 1.0, 2.0 and 4.0 kHz warble tones delivered to the four sites: two BC sites (mastoid; forehead), and two STC sites (under the chin-submental region; neck—over the SCM).

In order to efficiently change between the four stimulation sites and the two occlusion conditions, the order of stimulus presentation and of the occlusion condition was as follows: first the threshold to mastoid stimulation was determined unoccluded, then the earplug was inserted and threshold of both mastoid and forehead stimulation was measured in the occluded condition; after that the earplug was removed and the thresholds to forehead and chin in the unoccluded condition were measured, followed by a second insertion of the earplug for the determination of both chin and neck occluded thresholds. Finally, after removing the earplug for the second time, the threshold at the unoccluded neck site was measured. The duration of a recording session in a typical subject was about 20 min. In this way, the occluded and unoccluded thresholds were determined without moving the elastic band during stimulation at a site, and were assessed with an identical application force.

The magnitude of the OE was calculated as the difference between the open unoccluded threshold and the occluded threshold for each frequency, for each site and for each participant. The results were analyzed statistically using 2-way ANOVA for the occlusion effect at the four different sites for the four frequencies.

The study was reviewed and approved by the Hebrew University—Hadassah Medical Center Ethics Committee, and written informed consent was obtained from all participants.

## 3. Results

**Figure 1:**
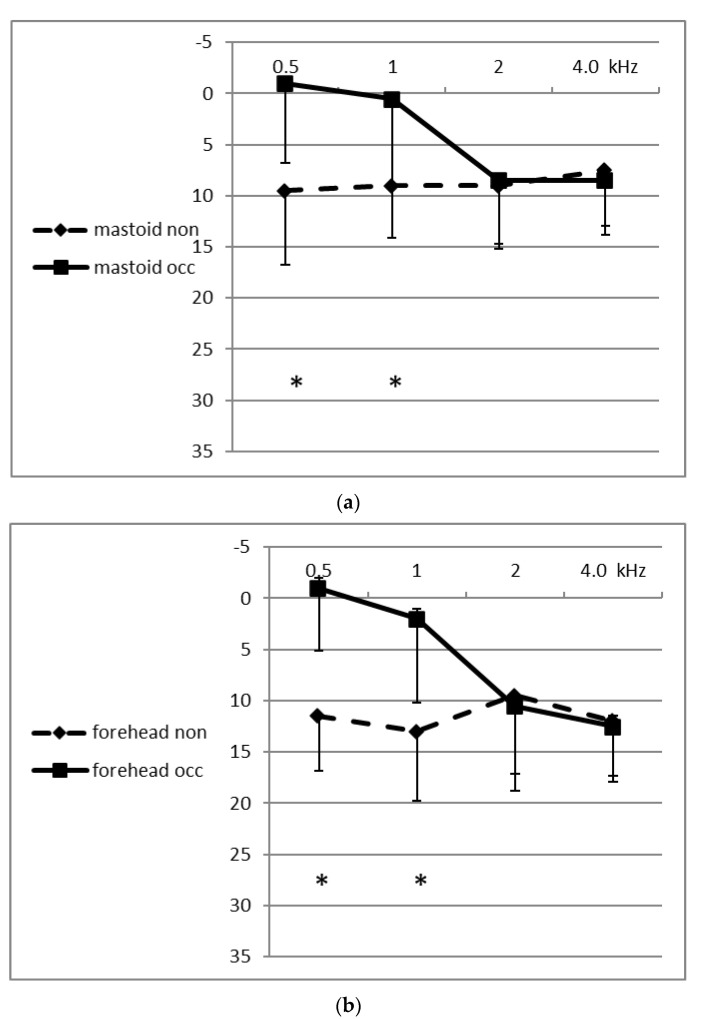
Mean thresholds (dB HL) and standard deviations in response to stimulation at the two bone conduction (BC) sites: (**a**) mastoid & (**b**) forehead when the external canal was non-occluded (dashed line) and occluded (continuous line). The vertical distance between the two data points for each frequency provides an approximate measure of the magnitude (dB) of the occlusion effect (OE). The numerical values of the mean OE (± SD) of the participants, at each frequency and for each site, are shown in the table. The * indicates significant difference between occluded and non-occluded thresholds.

**Figure 2:**
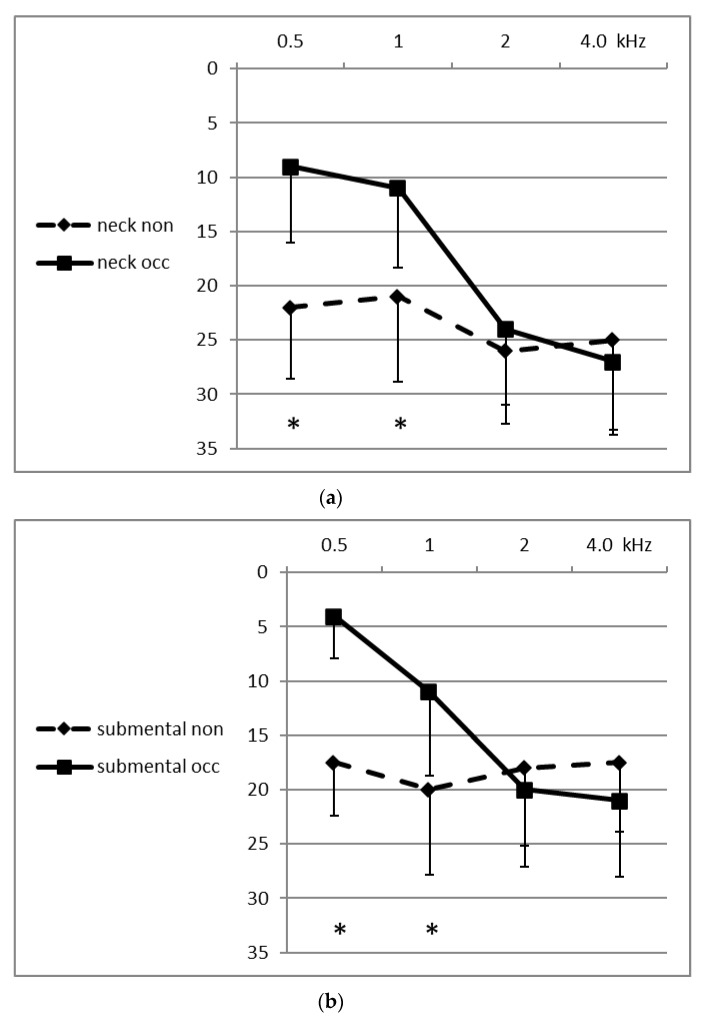
Mean thresholds (dB HL) and standard deviations in response to stimulation at the two STC sites: (**a**) neck = over the sterno-cleido-mastoid muscle & (**b**) submental = under the chin; when the external canal was non-occluded (dashed line) and occluded (continuous line). The vertical distance between the two data points for each frequency provides an approximate measure of the magnitude (dB) of the OE. The numerical values of the mean OE (± SD) of the participants, at each frequency and for each site, are shown in the table. The * indicates significant difference between occluded and non-occluded thresholds.

Thresholds could be obtained from each of the 10 participants in response to stimuli delivered at 0.5, 1.0, 2.0 and 4.0 kHz to the two STC (submental and neck) sites, as well as to the two BC (mastoid and forehead) sites, in open and in occluded conditions. Figure 1 shows the means of the thresholds in dB HL of the BC sites in the open and occluded conditions for the four frequencies. Figure 2 shows the means of the thresholds for the STC sites. It can be seen in both figures that the occluded thresholds were lower (better) at the medium-lower frequencies (0.5 and 1.0 kHz) both at the BC sites and at the STC sites. The differences between the open and occluded thresholds were significant (two-way ANOVA; *p* < 0.001). The magnitude of the occlusion effect for each participant, at each frequency and at each site, was calculated by subtracting the occluded threshold from the unoccluded threshold. Table 1 displays the mean (±SD) values of the magnitude of the occlusion effect (unoccluded–occluded threshold differences) for all four frequencies at each of the sites. The OE at the medium-low frequencies reached a mean maximal magnitude of 13.5 dB at the chin (submental-STC) site at 0.5 kHz, and reached a minimal value of 6.5 dB at 1.0 kHz at the neck-STC site. The two-way ANOVA showed that there was a significant difference in the occlusion effect between the lower frequencies compared to the higher frequencies (*p* < 0.001), and that there was no significant difference between the occlusion effect for the two STC sites compared to the two BC sites. In fact, at the higher frequencies, the mean magnitude of the OE was negative in value (not significant) at each of the four sites and at 4.0 and 2.0 kHz at both of the STC (chin and neck) sites, meaning that the threshold in the open canal state was lower (better) at these sites and frequencies than when the external canal was occluded.

## 4. Discussion

This study has shown for the first time that the occlusion effect can be elicited in response to stimulation delivered to soft tissue sites (STC), not only to BC stimulation. Even though it is assumed that there is greater damping through the soft tissues, the OE was elicited in response to STC. The two-way ANOVA showed that the magnitude of the OE at the two STC sites was similar to that at the two BC sites. However, while the bone vibrator and audiometer had been calibrated to deliver vibratory stimuli to the rigid bony sites (mastoid and forehead) presenting higher mechanical impedance, the STC sites presented lower impedance. Therefore, the occluded and open threshold values at the two STC sites cannot be directly compared to the occluded and open threshold values at the two BC sites. However, the unoccluded and occluded threshold differences (i.e., the magnitude of the OE) at the two STC sites can be reliably compared statistically to those at the two BC sites, and this comparison showed that there was no difference between them.

It has been shown that there is a positive correlation between the magnitude of the OE to BC stimuli and the sound pressure level present in the occluded canal at the same time [3,5]. Therefore, it is likely that the OE in response to BC stimuli is a result of the sound pressures in the air entrapped in the canal when it is occluded. The magnitudes of OE to BC stimuli reported by others are generally similar to the BC OE found in the present study: greater at the lower frequencies; non-existent or negligible at the higher frequencies [3,5,6]. The same result was found in the present study for the OE in response to STC stimulation: greater at the lower frequencies; non-existent or negligible at the higher frequencies.

The OE found in the present study in response to STC stimulation was also likely related to the vibrations induced in the soft tissue-cartilage during STC stimulation; the vibrations not only reach and excite the inner ear, they also reach the more compliant soft tissue-cartilaginous wall of the external canal, and when it is occluded, the entrapped air pressures drive the tympanic membrane, ossicles, etc. Thus, the OE in response to STC stimulation is a logical consequence of the mechanisms of STC.

Why is the OE induced by STC stimulation, presented at threshold intensities, dominated by the lower frequencies? A similar relation between the magnitude of the OE in response to BC stimulation was found in the present study: a greater OE at the medium-lower frequencies; negligible at the medium-higher frequencies. In a study on cadavers [7], the workers found that removal of the soft tissues and cartilage parts of the external canal resulted in a reduced sound pressure in the occluded canal to BC stimulation. They therefore conclude from this that the soft tissues and cartilage parts of the external canal are responsible for the OE to BC stimulation at the lower frequencies [7,8]. Since the OE in response to both osseous BC and STC stimulation was found to be dominated by the lower frequencies, and non-existent or negligible at the higher frequencies, it is possible that the OE to both BC and STC stimulation is mainly due to the contribution of the vibrations of the more compliant soft tissue-cartilaginous parts of the external canal, while the more rigid inner bony portions of the external canal may not contribute to the OE at threshold intensities. This suggestion can be explained by the anatomy of the canal—the more compliant soft tissue-cartilaginous wall of the canal is present over a larger area of the canal than the more rigid inner bony part of the wall. Therefore, the vibrations of the more compliant soft tissues will produce higher entrapped air pressures in the occluded canal at the lower frequencies.

The involvement of the soft tissues of the wall of the canal in generating the OE is supported by recording the sound pressures in the canal resulting from the intrinsic sounds of the heartbeat and blood flow which reach the canal. The sound pressures were recorded with a miniature microphone inserted into the external canal of the participants, and the recorded signal was time-averaged for noise reduction, triggered by the carotid pulse detected by a vibrometer [9]. The difference between the sound pressures recorded when the canal was unoccluded (open) and occluded (the OE) was 40 dB at 40 Hz in all participants, and 50 dB at 20 Hz in some individuals [9]—i.e., much greater than the OE to the higher frequencies. In order for the sound pressure in the occluded canal to be elevated from 40 to 50 dB (the OE), the magnitude of the vibrations of the walls of the canal would have to be relatively large, and therefore is more likely due to vibrations of the more compliant soft tissue-cartilaginous wall, rather than that of the rigid bony part of the canal wall.

### Soft Tissue Conduction via Stethoscope

At this point, it is interesting to point out that in a 1949 article, von Bekesy [10] reported that the vibrations from the vocal cords during self-vocalization are distributed over the entire body, and he used a stethoscope to estimate the distribution of these vibrations on the surface of the body [10]. What did Bekesy do with a stethoscope? The stethoscope is a simple mechanical instrument. It is apparent that the use of the stethoscope enabled von Bekesy to detect the vibrations initiated by the vocal cords which reached the surface of the body through the soft tissues (i.e., STC) [10]. The vibrations detected by the diaphragm of the stethoscope on the body surface are transformed into air pressures which are conducted through the air in the tubing of the stethoscope (air conduction) to the listener’s ears. At the same time, the soft tissue vibrations reach the ear of the speaker, enabling him to hear his vocalizations by STC. Ascribing the hearing of one’s own voice to BC is a remnant of the time when all forms of hearing other than AC were called BC [10,11,12].

## 5. Conclusions

In addition, in the article referred to earlier in this report [9], the very low frequency OE studied was actually elicited in response to intrinsically generated body sounds—i.e., STC, resulting from the heart and blood flow of the participants themselves, and conducted by the soft tissues to the wall of the external meatus. In fact, with one of the tips used to occlude the meatus, most participants were able to hear their own heartbeat—i.e., the OE induced by this tip enhanced the magnitude of the sound pressure in the meatus, so that the participant was able to perceive his own heartbeat.

Similar to the use of a stethoscope by von Bekesy to assess vocalizations on the surface of the body [10], the internist uses the stethoscope to “listen” to inherent body sounds elicited by the heartbeat, blood flow, air flow to and from the lungs, and intestinal movements which reach the surface (skin) of the body of the patient via the soft tissues (i.e., STC) during their routine physical examination.

## 6. Final Statement

The use of the stethoscope to hear intrinsic spontaneous body sounds resulting from the heart beat and blood flow [9] of his patient by the internist, and those occurring during vocalizations, as studied by von Bekesy [10], and of the fetal heartbeat by the obstetrician, and furthermore, the hearing of maternal sounds by the fetus [13], all serve as clear demonstrations of soft tissue conduction and its relation to hearing. In addition, the presence of the OE in response to stimulation at soft tissue sites provides complimentary evidence of the nature of STC. Thus, while osseous BC is a somewhat artificial form of hearing induced by vibrations delivered by a special mechanical vibrator and used for diagnostic purposes or as a hearing aid (bone anchored hearing aid), STC is a more natural form of hearing, occurs spontaneously and intrinsically in the body and plays a key role in basic clinical diagnosis. The implications of this study should not be understood as suggesting that an STC test should be added to the routine test battery in audiological clinics. Rather, audiologists should be aware that several auditory phenomena are actually the result of STC; for example the occlusion effect as detailed in the present study, the hearing of one’s own voice and a fetus hearing in-utero maternal sounds [13]

## Figures and Tables

**Table 1:** Mean ± SD of the magnitudes of the OE (unoccluded–occluded threshold differences) across all of the participants in dB at each of the four stimulation sites, at each of the four frequencies. A negative value indicates that the threshold in the open canal state was lower (better) than that in the occluded state.

Frequency/Site	0.5 kHz	1.0 kHz	2.0 kHz	4.0 kHz	Low vs. High
mastoid	10.5 ± 5.5	8.0 ± 4.8	0.5 ± 1.6	−3.0 ± 5.3	*p* < 0.001
forehead	13.0 ± 3.5	11.0 ± 4.5	0.5 ± 3.7	−2.0 ± 2.6	*p* < 0.001
chin	13.5 ± 4.1	8.0 ± 5.4	−0.5 ± 2.8	−4.0 ± 2.1	*p* < 0.001
neck	10.5 ± 4.4	6.5 ± 5.8	−3.0 ± 3.5	−7.0 ± 4.8	*p* < 0.001
